# Effects of a Digitally Supported Physical Activity Intervention in Knee Osteoarthritis: A Pilot Randomized Controlled Trial

**DOI:** 10.1002/msc.70085

**Published:** 2025-03-27

**Authors:** Hakan Akgül, Murat Birtane, Eda Tonga

**Affiliations:** ^1^ Department of Physical Medicine and Rehabilitation Trakya University Faculty of Medicine Edirne Turkey; ^2^ Department of Physiotherapy and Rehabilitation Marmara University Faculty of Health Sciences İstanbul Turkey; ^3^ Diabetes Research Centre College of Life Sciences Department of Population Health Sciences University of Leicester Leicester UK; ^4^ NIHR Leicester Biomedical Research Centre Leicester UK

**Keywords:** digital health, exercise, knee, osteoarthritis, physical activity

## Abstract

**Objectives:**

The aim of this study was to investigate the effects of a digitally supported physical activity (PA) intervention on pain, physical function, exercise adherence and quality of life in females with knee osteoarthritis (OA).

**Methods:**

Thirty female participants with knee OA were randomly assigned to either a control group (*n* = 15) receiving patient education, smartwatch use, and a home exercise programme, or an intervention group (*n* = 15) receiving an additional digitally supported walking programme. The primary outcome measures were pain severity (Numerical Pain Rating Scale [NPRS]), physical function (Western Ontario and McMaster Universities Arthritis Index [WOMAC]) and exercise adherence. Secondary outcomes included quality of life (European Quality of Life Scale 5 Dimensions/EQ‐5D‐3L), daily step count and pain catastrophizing (Pain Catastrophizing Scale/PCS). Assessments were performed at baseline and after 8 weeks.

**Results:**

The intervention group showed significantly greater improvements in pain severity (1.4 cm larger improvement on NPRS; *p* = 0.005), physical function (6.4‐point larger improvement on WOMAC total score; *p* = 0.003) and daily step count (1548 steps larger improvement; *p* = 0.045) compared to the control group. Both groups demonstrated similar significant improvements in exercise adherence, pain catastrophizing, and quality of life (*p* < 0.05).

**Conclusions:**

A digitally supported PA intervention, in addition to patient education and a home exercise programme, significantly improved pain, physical function and PA in females with knee OA. These findings support the efficacy of structured, supervised digital interventions for enhancing outcomes in this population. Future studies should explore strategies to enhance long‐term adherence through digital PA interventions.

**Trial Registration:**

This study was conducted in compliance with the Declaration of Helsinki (Clinical Trial Registry Number: NCT05810376)

## 1 Introduction

Osteoarthritis (OA) is the most prevalent chronic joint disease, characterised by progressive cartilage degeneration, structural joint changes, and inflammation, that leads to pain and reduced mobility (Focht 2012; Lopes et al. 2023; Mesci et al. 2015). It is estimated that approximately 654 million people worldwide are affected by knee OA (Cui et al. 2020). Notably, a significant proportion of these patients are of active working age, with over half under the age of 65. The prevalence of knee OA is expected to rise in the future (Cross et al. 2014; Deshpande et al. 2016). Individuals with knee OA often experience reduced functional capacity and quality of life, primarily due to loss of strength and physical inactivity stemming from pain or fatigue.

The World Health Organization defines physical activity (PA) as any body movement produced by skeletal muscles that requires energy expenditure (WHO 2019). Both moderate and vigorous PA have positive health effects, with common activities including walking, cycling, and sports (WHO 2019). Regular PA improves cardiovascular fitness and reduces the risk of chronic diseases. Current guidelines recommend at least 150 min of PA per week for all adults. PA is increasingly promoted for individuals with arthritis, as it is well‐established that it enhances health outcomes in people with OA. EULAR recommends regular PA as an integral component of OA management, with individualised objectives and assessment considering general and disease‐specific barriers and facilitators (Osthoff et al. 2018) Guidelines also recommend PA to improve pain and function in knee OA patients, regardless of disease severity; however, many patients still do not engage in sufficient PA (Dunlop et al. 2011; Zhang et al. 2010; Zhang et al. 2008). For these recommendations to be effective, clinicians must understand how to address PA barriers in patients with OA, which involve a complex interplay of pain, beliefs, and activity. The primary barriers to PA and exercise in OA patients are fear of movement and fear of exacerbating pain. Therefore, addressing these fears is crucial to preventing physical inactivity and enhancing adherence to regular exercise (Heuts et al. 2004; Somers et al. 2009). Some studies have successfully employed behavioural change techniques to enhance adherence with walking programs for individuals with knee OA (K. Bennell et al., 2020; Kim L Bennell et al., 2017; Wallis et al. 2017).

Digital health interventions offer a promising solution to physical inactivity as they can reach a broad segment of the population and are delivered efficiently at a low cost (Iribarren et al. 2017). These interventions can encompass patient education, self‐management support, motivation, follow‐up, feedback and communication, which may prevent, improve or treat various chronic conditions (Kardan et al. 2024; Zangger et al. 2023). Such features can boost patient motivation and encourage adherence to home exercise and PA (Meier et al. 2013). Although existing literature has explored digitally supported PA interventions and behaviour change techniques (Kim L Bennell et al. 2017; Li et al. 2020; Wallis et al. 2017), the evidence is still insufficient.

Cultural, environmental, and economic factors influence PA management. In Turkey, no studies have examined digital interventions or walking programs using behavioural change techniques for knee OA. Therefore, the aim of this study was to examine the effects of a digitally supported PA intervention on pain, functionality, and exercise adherence in individuals with knee OA.

## 2 Methods

### 2.1 Study Design

This study was designed as a two‐arm, parallel‐group, randomized controlled study and reported in accordance with CONSORT (Consolidated Standards of Reporting Trials) and CONSORT‐EHEALTH (Consolidated Standards of Reporting Trials of Electronic and Mobile Health Applications and Online TeleHealth) standards. The study took place at Trakya University Education and Research Hospital between October 2022 and August 2024.

Females diagnosed with knee OA by a physical medicine and rehabilitation specialist were invited to the study by the researcher via a telephone call. Individuals who met the inclusion criteria and agreed to participate were included in the study and randomized to the control group which received patient education, smartwatch use and home exercise programme or the intervention group which received digitally supported PA intervention based on social cognitive theory in addition to control interventions.

### 2.2 Ethics

Ethical approval was obtained from the Scientific Research Ethics Committee of the Faculty of Medicine of Trakya University (approval date and number: 2021/291). Informed consent was obtained from each participant before enrolment.

### 2.3 Participants

The inclusion and exclusion criteria for this study are outlined in Table 1.

**TABLE 1:** Inclusion and exclusion criteria of the study.

Participants were eligible if they: Were females aged 50 years or older. Had a diagnosis of knee OA based on the American College of Rheumatology criteria. Experienced pain or discomfort lasting more than 28 consecutive days in the past year. Used WhatsApp and had daily internet access.	Participants were excluded if they: Had a diagnosis of another rheumatic disease (e.g., rheumatoid arthritis, psoriatic arthritis, ankylosing spondylitis). Had a history of using antirheumatic drugs. Had undergone knee replacement surgery. Were on the waiting list for total knee or hip replacement surgery. Had surgery on the lumbar, hip, knee, foot, or ankle joints in the past 12 months.

### 2.4 Randomisation

To maintain allocation concealment, randomisation was carried out by a researcher not involved in the study and opaque and sealed envelopes were utilised. These envelopes were numbered sequentially and included assignments for each group. Once the baseline assessment was completed, the envelope was opened by the researcher and the assigned group was disclosed. Participants were randomized in a 1:1 ratio after collecting baseline data. Block randomisation was implemented to ensure that both groups remained comparable in sample size throughout the study.

### 2.5 Outcomes

The primary outcome measures of this study were as follows. (i) Pain severity measured by the Numerical Pain Rating Scale (NPRS), with scores ranging from 0 to 10, with higher scores indicating worse pain. A 1‐point improvement in NPRS score is considered as minimal clinically important difference (MCID) (Salaffi et al. 2004). (ii) Physical function measured by The Western Ontario and McMaster Universities Arthritis Index (WOMAC) with scores ranging from 0 to 100. The scale comprises three domains: pain, stiffness, and difficulty in daily activities. Higher WOMAC scores indicate greater stiffness, pain, and poorer physical function. A 10‐point improvement in total WOMAC score was considered as MCID (Clement et al. 2018). (iii) Exercise adherence was recorded via an exercise diary. The participants recorded the number of steps she regularly took every day and the number of exercises she performed. The patients were asked to record in their diaries any discomfort they experienced during daily life, walking or exercise. The secondary outcome measures were as follows. (i) Quality of life was measured using the EuroQoL (EQ‐5D‐3L) questionnaire, including the overall health for the day by a 0–100 visual analogue scale (VAS), and the EQ‐5D index value, which is a summary number reflecting a health state compared with the general population. (ii) Daily step counts were recorded using a valid and reliable smartwatch (Xiaomi Mi Band 3 Smartwatch, Xiaomi Technology Co. Ltd, Beijing) provided to each participant, ensuring consistent and objective measurement of PA levels (Hoogkamer et al. 2018). (iii) Pain catastrophizing was measured by the Pain Catastrophizing Scale (PCS) to assess participants’ catastrophic thinking related to pain, with scores ranging from 0 to 52, where higher scores indicate greater pain catastrophizing (Sullivan 2009).

### 2.6 Data Collection

Data on demographic information and eligibility to participate in the study were collected before the first week of smartwatch use (Week 0). Each participant was given a smartwatch 1 week before the intervention that would be used to measure the number of steps and was informed about how to use the smartwatch. The information included the usage of smartwatches, covering topics such as installation, operation, key considerations, and a question‐and‐answer section. Data were collected via the application integrated with the Xiaomi Mi Band 3 that the participants downloaded to their mobile phones and by recording their daily step counts. After 1 week of smartwatch use, all assessments were performed before the intervention (Week 1). A daily step count was recorded for each week during intervention. Participants used the smartwatches for at least 10 h per day to ensure valid measurement. At the end of each week, there had to be at least 4 days of valid measurement recorded (Hoogkamer et al. 2018). In order to prevent data loss, participants were asked to read and note their daily step counts from their smartwatches before going to bed. All assessments were repeated at the end of the intervention (Week 9).

**FIGURE 1:**
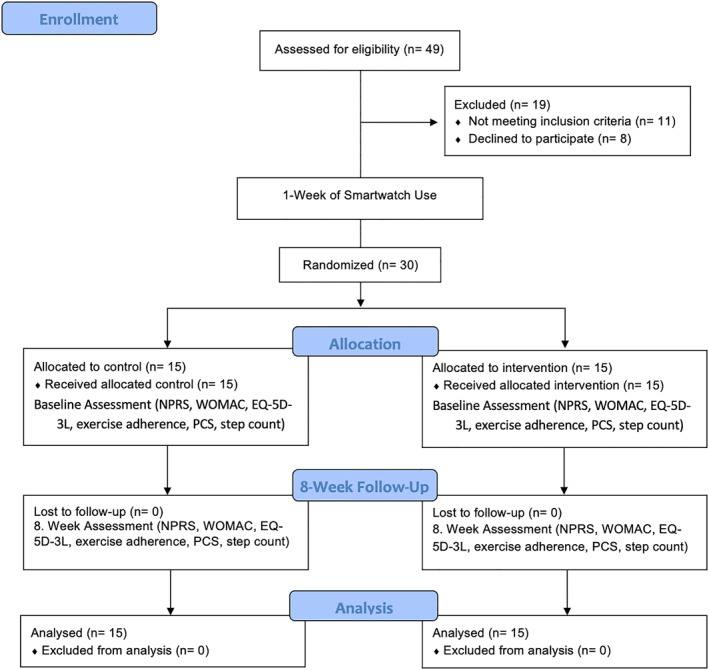
Flowchart.

The flowchart of the study is presented in Figure 1.

### 2.7 Interventions

#### 2.7.1 Patient Education

Participants in both groups received a 1‐h face to face education session by the researcher after the baseline assessments. The content of this education session was organised according to the PA guidelines (Nelson et al. 2014; WMRNGea Zhang et al. 2008). The control group only received the standard education session. The intervention group received additional digitally supported walking programme recommendations.

The content of the education session covered understanding OA, explaining its pain mechanisms, long‐term treatment management, the importance of activity modification and weight loss, supporting a healthy lifestyle, implementing exercises, recommending at least 70 min of moderate walking weekly in sessions of at least 10 min, encouraging walking at least 6000 steps daily according to previous recommendations (White et al. 2014) and explaining the use of smartwatches for tracking activity.

#### 2.7.2 Home Exercise Programme

The exercise programme included warm up, stretching (gastrocnemius, soleus and quadriceps muscles), and strengthening (Mini squat, terminal knee extension, hamstring and gastrocnemius strengthening) exercises. For warm‐up, 5–10 min of low‐impact activities such as riding a stationary bike or walking before starting the exercise as recommended to participants. The programme was applied in the order of exercise types as warm‐up, stretching, strengthening, stretching. The details of the exercise programme and instruction material given to participants can be found in Supporting Information S1. Both groups received the same exercise programme and performed the programme 3 days a week, 3 sets × 10 repetitions on each day for 8 weeks (Nixon 2018). Patients were asked to fill in the exercise diaries given to them every day for 8 weeks.

#### 2.7.3 Digital‐Based Walking Programme

A previous qualitative study was conducted by the researchers to explore the barriers and facilitators to exercise adherence in patients with knee OA from the perspectives of physiotherapists and patients (Akgül et al. 2020). Data were collected through focus group discussions with eight physiotherapists and semi‐structured interviews with five patients with OA. Thematic analysis identified key barriers such as limited beliefs in exercise benefits, inadequate patient education, fear‐avoidance beliefs, fatigue and poor self‐management. Both physiotherapists and patients highlighted the importance of patient education in improving adherence. Facilitators included motivational strategies from therapists, personalised exercise programs, and the use of digital technology.

The information collected from this study was synthesised from the literature and an 8‐week digital‐based walking programme planned to be implemented in the experimental group was created.

### 2.8 Intervention Protocol

The intervention aimed to gradually increase walking duration for participants based on their initial capacity and barriers related to pain or other symptoms. The first goal was to achieve *70 min of walking per week* for participants with limited walking ability. For those who could already manage 70 min, the goal was to *progress to 150 min per week*. Walking duration was adjusted weekly in collaboration with each participant, considering their discomfort and preferences.

#### 2.8.1 Weeks 1–3: Progression to 70 Minutes per Week

Week 1:

- Participants completed two sessions of 25‐min walks (total 50 min per week).
- A 5‐min video call via WhatsApp was conducted before and after each walk.

Week 2:

- Walk duration increased to two sessions of 30 min (total 60 min per week).
- A 5‐min video call via WhatsApp was conducted before and after each walk.
- A WhatsApp message was sent before and after the second walk to update participants on their progress.

Weeks 3–8:

- Walk duration increased to two sessions of 35 min per week (total 70 min per week).
- Participants received WhatsApp messages for continued support and motivation.

#### 2.8.2 Weeks 4–8: Progression Beyond 70 Minutes

For participants who could walk more than 70 min per week, the following progression was followed:

Weeks 4–5:

- Two sessions of 35‐min walks (total 70 min per week).
- A WhatsApp message was sent before and after each walk.

Week 5:

- Walk duration increased to three sessions of 30 min (total 90 min per week).
- A WhatsApp message was sent before and after each walk.

Week 6:

- Walk duration increased to three sessions of 35 min (total 105 min per week).
- A WhatsApp message was sent before and after each walk.

Week 7:

- Walk duration increased to three sessions of 40 min (total 120 min per week).
- A WhatsApp message was sent before and after each walk.

Week 8:

- Walk duration increased to three sessions of 50 min (total 150 min per week).
- A WhatsApp message was sent before and after each walk.

This structured approach ensured gradual progression while accommodating individual limitations and preferences, promoting adherence and minimising discomfort.

To increase compliance with the intervention, the following behavioural change techniques and strategies based on the Social Cognitive Theory were implemented. Firstly, each participant made a plan with the researcher via WhatsApp application to plan the place, day and time for each walk and to reinforce that each walk would be at least moderate in intensity for a period of 10 min (Goal Setting and Self‐Efficacy). Secondly, regular physiotherapy supervision and follow‐up were conducted every week, including one‐on‐one supervised walking sessions (such as video calls, sending photos via WhatsApp application) according to the participant’s preferred time and regular phone calls or SMS reminders (Reinforcement). Thirdly, each participant used the smartwatch given to them and recorded the number of steps taken in each session in a diary (Self‐Monitoring). Lastly, participants were encouraged to use social supports, such as walking with a friend, family member, or other research participants if they wished (Social Support) (Greaves et al. 2011; Wallis et al. 2017).

### 2.9 Statistical Analysis

Mean and standard deviations (SD) for continuous variables and frequencies for categorical variables were used to present descriptive analyses. Exercise adherence was classified as yes (participants who followed the exercise programme at least 3 days on that week) or no (participants who did not follow the exercise programme at least 3 days on that week) for each week from baseline to follow‐up. Adherence was reported as the number of weeks with adherence to the exercise programme from baseline to follow‐up and this Mann–Whitney U test was used to compare the exercise adherence between control and intervention groups. Repeated measures ANOVA was used to analyse the effect of time and group × time interaction on the dependent variables which were NPRS, WOMAC, EQ‐5D‐3L, step count and PCS scores. Independent variables were the control or intervention groups. The level of significance was set at *p* < 0.05. All analyses were performed using IBM SPSS Statistics version 22. The observed power of the study was calculated to be 85% based on the effect size of NPRS (partial eta squared: 0.254) with a sample size of 30 participants at the 5% level of significance.

## 3 Results

### 3.1 Participant Characteristics

A total of 30 female participants were randomly assigned to either the control group (*n* = 15) or the intervention group (*n* = 15). Table 2 presents the characteristics of the participants. The mean age of the participants was 62.1 ± 8.6 years for the control group and 58.7 ± 5.2 years for the intervention group. No significant differences were observed between groups in terms of baseline characteristics (*p* > 0.05), with the exception of step count at week 0 (*p* = 0.009).

**TABLE 2:** Demographic and clinical characteristics of the participants by groups.

	Control group (*n* = 15) (mean [SD]) or (*n* [%])	Intervention group (*n* = 15) (mean [SD]) or (*n* [%])
Age	62.1 (8.6)	58.7 (5.2)
BMI (kg/m^2^)	33.3 (6.7)	29.1 (6.3)
Baseline pain (NPRS)	3.8 (1.7)	4.1 (2.5)
Week 0 step count (steps per week)^a^	3992.4 (1197.1)	6056.7 (3147.6)
Education		
Primary school	2 (13.3)	3 (20.0)
Secondary school	2 (13.3)	3 (20.0)
High school	8 (53.3)	6 (40.0)
Undergraduate or higher	3 (20.0)	3 (20.0)

Abbreviations: BMI, body mass index; NPRS, numerical pain rating scale; SD, standard deviation.

^a^ Data on the first week of step count was collected before the baseline assessments (Week 0).

### 3.2 Pain

**FIGURE 2:**
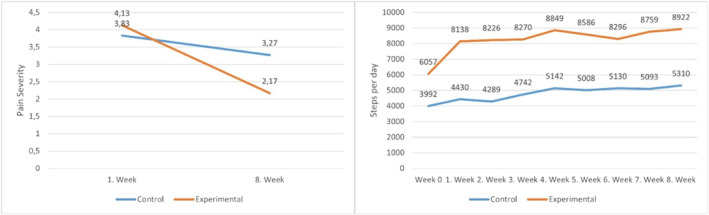
Outcomes on NPRS and step count from baseline to 8‐week follow‐up for control and intervention groups.

The NPRS scores demonstrated a significant time effect (*p* < 0.001) and a significant group‐time interaction (*p* = 0.005, partial eta squared = 0.254). From baseline to 8‐week follow‐up, the average difference in the change in NPRS scores between groups was 1.4 (95% CI: 0.5–2.3) favouring the intervention group, respectively (Table 3, Figure 2). A MCID was achieved by 33.3% (*n* = 5) of participants in the control group and 80.0% (*n* = 12) in the intervention group. MCID analysis indicated that a higher proportion of participants in the intervention group achieved clinically meaningful improvements in NPRS scores compared to the control group (Table 4).

**TABLE 3:** Descriptive data of outcome measures for each time and group, and mean difference between groups from baseline to 8‐week follow‐up.

Time‐points	Control group mean (SD)	Intervention group mean (SD)	Difference in mean change (95% CI)	Time effect *p*‐value	Time × group interaction *p*‐value	Effect size (partial eta squared)
NPRS						
Baseline	3.8 (1.7)	4.1 (2.5)	1.4 (0.5 to 2.3)	**< 0.001**	**0.005**	0.254
8. Week	3.3 (1.5)	2.2 (1.1)				
WOMAC total						
Baseline	35.3 (13.0)	29.1 (13.2)	6.4 (2.4 to 10.4)	**< 0.001**	**0.003**	0.280
8. Week	32.1 (12.3)	19.5 (9.3)				
WOMAC pain						
Baseline	6.2 (2.8)	6.0 (3.7)	2.1 (0.1 to 4.2)	**< 0.001**	**0.043**	0.138
8. Week	5.1 (2.6)	2.8 (1.8)				
WOMAC stiffness						
Baseline	2.5 (1.5)	2.7 (1.9)	0.8 (−0.1 to 1.7)	**0.004**	0.070	0.112
8. Week	2.3 (1.5)	1.6 (1.0)				
WOMAC function						
Baseline	26.6 (10.5)	20.5 (10.1)	3.5 (0.8 to 6.1)	**< 0.001**	**0.011**	0.207
8. Week	24.7 (10.1)	15.1 (7.9)				
EQ‐5D‐3L						
Baseline	0.50 (0.23)	0.59 (0.20)	0.01 (−0.09 to 0.10)	**0.004**	0.834	0.002
8. Week	0.56 (0.17)	0.66 (0.18)				
Step count						
Week 0^a^	3992.4 (1197.1)	6056.7 (3147.6)	1548.0 (323.6 to 2772.4)	**< 0.001**	**0.045**	0.487
8. Week	5309.7 (1639.4)	8922.0 (2863.2)				
PCS						
Baseline	12.1 (8.9)	15.5 (9.2)	1.8 (−1.0 to 4.6)	**< 0.001**	0.201	0.058
8. Week	9.6 (7.6)	11.2 (7.0)				

Abbreviations: CI, confidence interval; EQ‐5D‐3L, European quality of life scale 5 dimensions 3 levels; NPRS, numerical pain rating scale; PCS, pain catastrophizing scale; SD, standard deviation; WOMAC, Western Ontario and McMaster Universities Arthritis Index.

^a^ Data on the first week of step count was collected before the baseline assessments (Week 0).

**TABLE 4:** Minimal clinically important differences by groups from baseline to 8‐week follow‐up.

	Control group *n* (%)	Intervention group *n* (%)
NPRS	5 (33.3)	12 (80.0)
WOMAC	0 (0.0)	6 (40.0)

Abbreviations: NPRS, numerical pain rating scale; WOMAC, Western Ontario and McMaster Universities Arthritis Index.

### 3.3 Physical Function

Physical function, assessed using the WOMAC scale, showed a significant time effect (*p* < 0.001) and a significant group‐time interaction (*p* < 0.05) for WOMAC total scores (partial eta squared: 0.280) and pain (partial eta squared: 0.138) and function (partial eta squared: 0.207) subscores. For WOMAC stiffness, there was a significant time effect (*p* = 0.004) but no significant group‐time interaction (*p* = 0.070, partial eta squared = 0.112) (Table 3). Additionally, 40.0% (*n* = 6) of participants in the intervention group achieved an MCID in WOMAC scores, whereas none in the control group did. MCID analysis indicated that a higher proportion of participants in the intervention group achieved clinically meaningful improvements in WOMAC scores compared to the control group (Table 4).

### 3.4 Exercise Adherence

**FIGURE 3:**
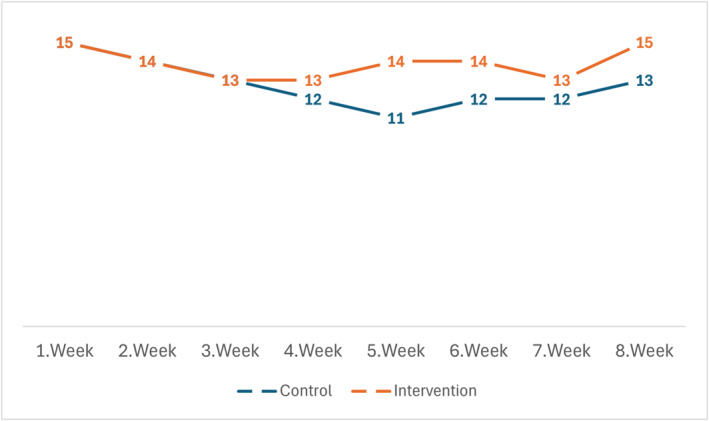
Number of participants adhered to the exercise programme at least 3 days per week for control and intervention groups.

Exercise adherence was high in both groups. In the control group, 11 participants (73.3%) adhered to the exercise programme at least 3 days per week, showing 100% adherence. In the intervention group, 12 participants (80.0%) exhibited full adherence. No statistically significant difference was observed between the groups regarding adherence (*p* > 0.05) (Figure 3).

### 3.5 Quality of Life

Quality of life, measured using the EQ‐5D‐3L, showed a significant time effect (*p* = 0.004) but no significant group‐time interaction (*p* = 0.834, partial eta squared = 0.002). The control group improved from 0.50 ± 0.23 at baseline to 0.56 ± 0.17 at 8 weeks, while the intervention group improved from 0.59 ± 0.20 to 0.66 ± 0.18 (Table 3).

### 3.6 Step Count

Step count showed a significant time effect (*p* < 0.001) and a significant group‐time interaction (*p* = 0.045, partial eta squared = 0.487). The baseline step count was significantly higher in the intervention group (6056.7 ± 3147.6 steps) compared to the control group (3992.4 ± 1197.1 steps, *p* = 0.009) (Table 2). From baseline to 8‐week follow‐up, the average difference in the change in step count between groups was 1548.0 (95% CI: 323.6–2772.4) steps favouring the intervention group, respectively (Table 3, Figure 2).

### 3.7 Pain Catastrophizing

Pain Catastrophizing Scale (PCS) scores showed a significant time effect (*p* < 0.001) but no significant group‐time interaction (*p* = 0.201, partial eta squared = 0.058). PCS scores decreased from 12.1 ± 8.9 to 9.6 ± 7.6 in the control group and from 15.5 ± 9.2 to 11.2 ± 7.0 in the intervention group (Table 3).

The results of NPRS and step count measurements for the control and intervention groups from baseline to 8‐week follow‐up are presented in Figure 2.

#### 3.7.1 Weekly Walking and Communication Plan Outcomes

All participants aimed to walk for at least 70 min over the course of 8 weeks. Digital communication, including video calls, text messages, and photo messages, is summarised in Table 5.

**TABLE 5:** Weekly walking and communication plan.

Week	Walking (twice a week)	Video call (before & after walk)	Message (before & after walk)	Photo (step count & exercise log)
1	25 + 25 = 50 min	1st walk: 5 + 5 = 10 min	None	Monday
		2nd walk: 5 + 5 = 10 min		
2	30 + 30 = 60 min	1st walk: 5 + 5 = 10 min	2nd walk	Monday
3–8	35 + 35 = 70 min	None	1st walk and 2nd walk	Monday

## 4 Discussion

### 4.1 Summary of Findings

This study explored the impact of a digitally supported PA intervention, alongside patient education and a home exercise programme, on key clinical outcomes in females with knee OA. Findings indicate that participants who received digital support in addition to standard education demonstrated significantly greater improvements in pain severity, physical function and daily step counts compared with those in the control group. However, both groups achieved comparable gains in exercise adherence, pain catastrophizing and quality of life.

### 4.2 Pain

Increasing PA is challenging for most individuals, and this difficulty is further amplified in those with knee OA (Kanavaki et al. 2017). Among the various barriers to PA, joint pain is frequently cited as a significant obstacle that limits mobility, reduces motivation, and impacts overall adherence to exercise programs (Kanavaki et al. 2017). However, despite these challenges, PA has been shown to play a crucial role in pain management for individuals with knee OA (Osthoff et al. 2018). Consistent with these findings, our study also demonstrated that increasing PA through a structured intervention was associated with a reduction in pain levels.

In our study, the intervention group experienced a mean reduction of 1.9 points in NPRS scores from baseline to 8 weeks, whereas the control group showed a smaller reduction of 0.5 points. These findings align with previous research, including Hinman et al., who examined a personalised behaviour change intervention focused on strengthening and PA (Hinman et al. 2020). Their study reported a significant decrease in pain, with a 1.8‐point reduction in the control group and a 2.5‐point reduction in the intervention group. PA is often perceived as a source of pain. However, as seen from our results, PA can help alleviate pain when combined with joint protection and PA modifications.

Consistent with the improvements observed in NPRS scores, the intervention group demonstrated a greater reduction in pain, as reflected in the WOMAC pain subscale, with a 3.2‐point decrease. This suggests that the digital PA intervention had a meaningful impact on alleviating pain and enhancing functional activity. These findings align with previous research highlighting the role of exercise in improving pain perception and functional outcomes. For instance, a study of 182 patients with knee OA comparing PA management via phone calls to usual care alone reported a significant 1.6‐point reduction in WOMAC pain scores in the intervention group, compared to a 0.3‐point decrease in the control group (Schlenk et al. 2021). Additionally, other digital PA interventions, including app‐based and web‐supported programs, have demonstrated similar benefits in reducing musculoskeletal pain and improving mobility, particularly when incorporating behavioural change strategies such as goal setting, personalised feedback and remote support (Li et al. 2020; Stevenson et al. 2024; Wallis et al. 2017).

### 4.3 Physical Function

Increasing physical function is a key goal in the management of knee OA, as impaired mobility and reduced daily activity levels significantly affect the quality of life in this population (Raposo et al. 2021). Similar to the barriers faced in increasing PA, factors such as joint pain, stiffness and decreased muscle strength can limit functional performance (Sattler et al. 2023). Despite these challenges, structured exercise programs have consistently been shown to improve physical function by enhancing joint stability, muscle strength and movement efficiency (Rocha et al. 2020). Consistent with these findings, our study demonstrated that participation in a structured intervention led to notable improvements in physical function, highlighting the positive impact of targeted PA strategies on functional outcomes in individuals with knee OA.

The improvements observed in physical function, as reflected in the WOMAC function subscale, align with previous research supporting the benefits of structured exercise programs for knee OA. Our findings are comparable to those of Schlenk et al., who reported similar functional gains following a self‐efficacy‐based PA intervention (Schlenk et al. 2021). The relatively smaller improvement in the control group is consistent with studies where less structured or targeted interventions resulted in more modest functional benefits, reinforcing the importance of supervised PA programs. However, studies by Nelligan et al. (2021) and Hinman et al. (2020) demonstrated greater improvements with functional gains ranging from 10.4 to 10.9 points. This may be attributed to their incorporation of personalised behaviour change strategies alongside PA interventions, suggesting that tailored approaches may further enhance functional outcomes.

In terms of stiffness, although the improvements were smaller compared to the pain and function subscales, the intervention group still experienced a meaningful reduction in perceived stiffness. Limited evidence is available on WOMAC stiffness outcomes, with Wallis et al. reporting minimal or even negative changes despite a similar intervention approach (Wallis et al. 2017). Differences in exercise protocols used in usual care across studies may have influenced these variations, highlighting the need for further investigation into how different PA components impact stiffness outcomes. Future research should explore the integration of personalised strategies to optimise functional and symptomatic improvements in knee OA management.

### 4.4 Daily Step Count

Promoting daily PA is a critical component in the management of knee OA, as maintaining an active lifestyle helps improve joint health, reduce pain and enhance overall function. However, increasing daily activity levels can be challenging due to factors such as pain, fatigue, and reduced motivation (Xiang et al. 2024). Despite these barriers, structured PA (PA) interventions, particularly those incorporating digital tools and behavioural strategies, have shown promise in encouraging sustained activity (Xiang et al. 2024). Consistent with this evidence, our study demonstrated that a structured digital PA intervention led to a significant increase in daily step count, emphasising the potential of targeted, technology‐supported approaches to effectively promote PA among individuals with knee OA.

The increase in daily step count observed in the intervention group suggests that a structured digital PA intervention can effectively promote PA among individuals with knee OA. Our findings indicate a greater increase in step count compared with previous studies. For instance, Wallis et al. reported only a modest 161‐step increase, while Linda et al. found an 839.3‐step improvement following an intervention combining patient education, a mobile app and motivational phone calls (Li et al. 2020; Wallis et al. 2017). Similarly, Stevenson et al. observed a 494‐step increase in a study utilising a 12‐week personalised PA programme delivered through an intelligent mobile application (Stevenson et al. 2024). In contrast, Bell et al. found no significant improvement in daily step count when using motivational interviewing alongside neuromuscular exercise and patient education (Bell et al. 2023).

The relatively higher step count increase in our study may be attributed to multiple factors, including time of the recruitment (summer), the more intensive and personalised nature of the intervention (e.g., motivational behaviour change techniques such as reminders, motivational talks and messages) and its shorter duration of 8 weeks, which may have contributed to greater engagement. Additionally, the lower baseline step counts in our participants compared with those in other studies may have allowed for a greater potential for improvement. These findings highlight the effectiveness of targeted PA interventions in promoting activity levels, but future research should explore the sustainability of these increases and the role of behavioural strategies in maintaining long‐term engagement.

### 4.5 Quality of Life and Pain Catastrophizing

Enhancing the quality of life is a fundamental goal in the management of knee OA, as individuals often experience limitations not only in physical function but also in psychological well‐being and overall life satisfaction (Xiang et al. 2024). While PA interventions are well‐documented for their positive effects on pain and mobility, their influence on broader quality of life outcomes can vary due to the complex interplay of physical, emotional and social factors (Xiang et al. 2024). Despite these challenges, structured PA programs have demonstrated the potential to improve the quality of life through both direct physical benefits and indirect psychological gains (Lopes et al. 2023). In line with this, our study observed improvements in quality of life measures, highlighting the role of targeted interventions in addressing the multifaceted impact of knee OA.

Both the control and intervention groups demonstrated similar improvements in quality of life, with only marginal differences in EQ‐5D scores (0.06 and 0.07 points, respectively). These findings align with previous research, such as Wallis et al., who reported a 0.09‐point increase in EQ‐5D following a 12‐week walking programme, compared to a slight decline in the control group receiving usual care (Wallis et al. 2017). Similarly, Bell et al. found a 0.10‐point improvement in an intervention incorporating motivational sessions alongside patient education and neuromuscular exercise, whereas the control group, receiving only patient education and neuromuscular exercise, showed a minor decline (Bell et al. 2023). These results reinforce the positive impact of structured and personalised PA interventions on the quality of life in individuals with knee OA, highlighting the need for their integration into standard treatment plans.

Despite these benefits, no significant between‐group differences were observed in pain catastrophizing, although both groups showed reductions over time (2‐point decrease in the control group and 4.3‐point decrease in the intervention group). In contrast, Bennell et al. reported greater improvements (6.2‐ and 7.0‐point reductions) following a 12‐week programme focused on pain coping skills, either alone or in combination with strengthening exercises (K. L. Bennell et al. 2016). This suggests that while our intervention was effective in reducing pain and function, its impact on psychosocial outcomes such as pain catastrophizing may have been more limited. Given that physical improvements are commonly achieved through exercise and behavioural interventions, addressing psychosocial factors may require more targeted psychological strategies or longer intervention durations to maximise benefits.

### 4.6 Exercise Adherence

Both groups in our study showed similar significant high adherence to the exercise programme, exercising at least 3 days per week. Our results are similar to those of other studies by Nelligan et al. (2021) and K. Bennell et al. (2020) who also reported high adherence both using behaviour change techniques via text messages. Despite strong overall adherence, several potential barriers may have influenced individual participation. Personal factors, such as fatigue, pain fluctuations, or competing responsibilities, could have affected consistency, particularly in later weeks. Economic constraints, including access to suitable exercise spaces or resources, may have also played a role, especially for individuals balancing work and caregiving duties. Additionally, psychological factors, such as self‐efficacy and perceived benefits of exercise, are known to impact adherence, as highlighted in prior research. While structured support within the intervention likely contributed to sustained engagement, addressing these barriers through tailored behavioural strategies may further enhance long‐term adherence and optimise intervention outcomes.

### 4.7 Limitations and Future Research

The primary limitations of our study arose during the patient recruitment phase, leading to a smaller‐than‐expected sample size. This was largely due to regional economic constraints, limited research funding, and insufficient researcher support. Additionally, as the first clinical physical activity intervention for arthritis patients in this region, the study may have been influenced by sociocultural factors that warrant further consideration.

Future research should examine sociocultural and economic barriers to physical activity participation in greater depth and establish both macro‐ and micro‐level support systems to improve accessibility. While our findings highlight the potential of digital PA interventions for pain management, further studies are needed to assess their long‐term effectiveness across diverse patient groups. Factors such as adherence, digital literacy, and individual responsiveness to remote guidance may significantly impact outcomes. Moreover, integrating interactive features, such as real‐time monitoring and AI‐driven coaching, could enhance engagement and maximise clinical benefits.

## 5 Conclusions

This study provides substantial evidence supporting the efficacy of a digitally supported PA intervention for improving pain, physical function, and PA in women with knee OA. The improvements in pain, physical function and PA and high adherence to exercise programme are consistent with previous studies and suggest that structured, supervised digitally supported PA intervention can significantly improve outcomes in females with knee OA. Future research should examine sociocultural and economic barriers to physical activity participation and identify solutions to improve accessibility and engagement. Additionally, further studies are needed to assess the long‐term effects of such interventions and explore the integration of behavioural strategies or advanced technology to enhance adherence and outcomes.

## Author Contributions

This study is part of the PhD thesis of Hakan Akgül supervised by Eda Tonga. The study was conceptualised and planned by Eda Tonga and Hakan Akgül. Murat Birtane supported patient recruitment, while data collection was carried out by Hakan Akgül. Analysis and interpretation of the data were performed by Hakan Akgül, Murat Birtane and Eda Tonga. The manuscript was drafted by Hakan Akgül and Eda Tonga. All authors critically revised the manuscript for important intellectual content and approved the final version for submission.

## Ethics Statement

Ethical approval was obtained from the Scientific Research Ethics Committee of the Faculty of Medicine of Trakya University (approval date and number: 2021/291).

## Consent

Informed consent was obtained from each participant before enrolment.

## Conflicts of Interest

The authors declare no conflicts of interest.

## Supporting information

Supporting Information S1

## Data Availability

The data that support the findings of this study are available on request from the corresponding author. The data is not publicly available due to privacy or ethical restrictions.
