# Supplementary material for: Effects of a Digitally Supported Physical Activity Intervention in Knee Osteoarthritis: A Pilot Randomized Controlled Trial
Source: Musculoskeletal Care. 2025 Mar 27;23(2):e70085. doi: 10.1002/msc.70085 (PMC11949769; doi:10.1002/msc.70085)
Supplement: Supplementary file 1 — Supporting Information S1 [file MSC-23-e70085-s001.docx]

**Home Exercise Program:**

- The exercises will be performed as a home exercise program three times a week, for 3 sets of 10 repetitions, over a period of 8 weeks.
- Patients will be asked to fill out the exercise logs provided to them every day for 8 weeks.

**Stretching Exercises;**

**1)** **Calf Stretching:** The patient stands facing a wall with the unaffected leg forward and slightly bent at the knee. The affected leg remains straight and positioned behind. Without lifting either heel off the ground, the patient leans forward towards the wall, feeling a stretch in the calf. This position is held for 30 seconds, followed by 30 seconds of rest, and then repeated.

**2)** **Quadriceps Stretch:** In a prone position (lying face down), the patient bends one knee. Using their hand or a sheet/belt, they pull their foot towards their glutes, increasing the knee flexion. The position is held for 5 seconds, followed by 5 seconds of rest, and then repeated.

**Strengthening Exercises;**

**3)** **Mini Squats:** The patient stands with feet shoulder-width apart and raises their arms to shoulder level. If there is a balance issue, they may use a chair or wall for support. While keeping the chest lifted, they perform a half squat, as if sitting on a chair. The position is held for 5 seconds, with the weight on the heels, and then the patient returns to the starting position.

**4)** **Hamstring Strengthening:** The patient stands behind a chair for support. The affected leg is bent at the knee, bringing the heel towards the ceiling, stopping at the pain threshold. The position is held for 5 seconds, followed by relaxation and a return to the starting position.

**5)** **Calf Strengthening:** The patient stands with weight evenly distributed on both feet and holds onto a chair for balance. The unaffected foot is lifted off the ground. The affected foot rises onto its toes as high as possible and then lowers. This movement is repeated 10 times.

**6)** **Leg Raise:** The patient sits on a chair or couch. By contracting the quadriceps (thigh muscles), they lift the leg upward. The position is held for 5 seconds, followed by relaxation and lowering the foot back to the floor. The movement is then repeated.
